# Errors in Byline and Affiliations

**DOI:** 10.1001/jamanetworkopen.2020.29182

**Published:** 2020-11-10

In the Consensus Statement titled, “Recommendations on Acute Kidney Injury Biomarkers From the Acute Disease Quality Initiative Consensus Conference: A Consensus Statement,”^1^ published October 6, 2020, there were errors in the byline and affiliations. Dr Legrand’s first name was misspelled, and Dr Joannidis’s affiliation should have read Division of Intensive Care and Emergency Medicine, Department of Internal Medicine, Medical University of Innsbruck, Innsbruck, Austria. This article has been corrected.^1^
